# *In vitro* and *in vivo* studies on bacteria and encrustation resistance of heparin/poly-L-lysine-Cu nanoparticles coating mediated by PDA for ureteral stent application

**DOI:** 10.1093/rb/rbac047

**Published:** 2022-07-06

**Authors:** Bukola O Awonusi, Jianzhong Li, Hongwei Li, Zhenyu Wang, Ke Yang, Jing Zhao

**Affiliations:** Shi-changxu Innovation Center for Advanced Materials, Institute of Metal Research, Chinese Academy of Sciences, Shenyang 110016, China; School of Materials Science and Engineering, University of Science and Technology of China, Hefei, 230026, China; Department of Urology, General Hospital of Northern Theater Command, Shenyang, 110840, China; Department of Urology, General Hospital of Northern Theater Command, Shenyang, 110840, China; Department of Urology, General Hospital of Northern Theater Command, Shenyang, 110840, China; Shi-changxu Innovation Center for Advanced Materials, Institute of Metal Research, Chinese Academy of Sciences, Shenyang 110016, China; Shi-changxu Innovation Center for Advanced Materials, Institute of Metal Research, Chinese Academy of Sciences, Shenyang 110016, China

**Keywords:** ureteral stents, nanoparticles, biofilm, encrustation

## Abstract

Ureteral stents are commonly utilized as a medical device to aid the flow of urine. However, biofilm formation and encrustation complications have been clinical problems. To overcome this challenge, heparin/poly-L-lysine-copper (Hep/PLL-Cu) nanoparticle was immobilized on a dopamine-coated polyurethane surface (PU/NPs). The stability and structural properties of the nanoparticles were characterized by Zeta potential, poly dispersion index, transmission electron microscopy, atom force microscopy and contact angle. The surface composition, antibacterial potency, encrustation resistance rate and biocompatibility of PU/NPs were investigated by scanning electron microscope, X-ray photoelectron spectroscopy, antibacterial assay and MTS assay, respectively. In addition, the anti-encrustation property was studied by implanting coated NPs stents in the rat bladder for 7 days. It was shown that the size and distribution of Hep/PLL-Cu nanoparticles were uniform. PU/NPs could inhibit *Proteus mirabilis* proliferation and biofilm formation, and exhibit no cytotoxicity. Less encrustation (Ca and Mg salt) was deposited both *in vitro* and *in vivo* on samples, demonstrating that the NPs coating could be a potential surface modification method of ureteral material for clinical use.

## Graphical Abstract

**Graphical Abstract:**
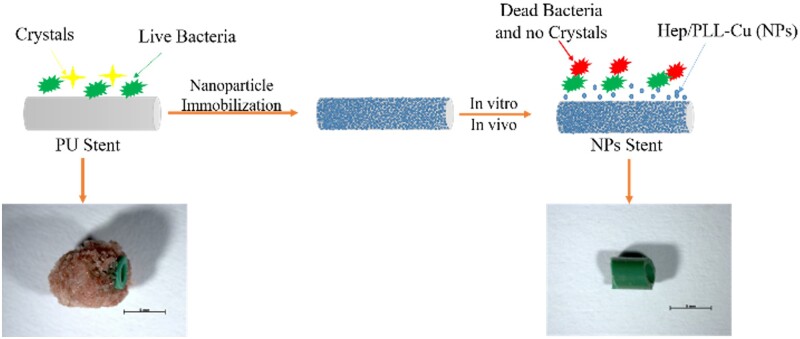

## Introduction

In the urological field, double J ureteral stents are one of the vital tools utilized for various clinical conditions, and as the manufacturing technique is upgrading, their applications have been expanded [1]. In the USA, about 92 000 ureteral stents, which are used to maintain urine drainage and to protect renal function, are placed annually to manage upper urinary tract obstructions caused by urolithiasis [2]. Polyurethanes (PU) are frequently applied for urological implants because of their good biocompatibility, and mechanical and physical properties [3–5].

However, infection and encrustation are significant problems that compromise the patients’ quality of life [6]. It was reported that the bacterial colonization rate on stents was 66.7% and 81.3% after implantation for 60–90 and 90–120 days. Besides, the incidence of encrustation with indwelling at 6, 6–12 weeks, and above 12 weeks was 9.2%, 47.5%, and even to be higher than 67.3%, respectively, which revealed that with increasing the indwelling time, the tendency of encrustation over double J stent increased critically [7–9]. Taking this into account, surface modification can be a candidate method for the practical use of new stent materials, which contributes to the inhibition of crystals and bacterial adhesion [10]. Wang *et al.* [11] demonstrated the antibacterial and anti-encrustation abilities of silver-containing polytetrafluoroethylene coated on silicone catheters. The polyethyleneimine brushes grafted on PU surfaces were also effective against biofilm formation and encrustation [6]. In a study by Francesko *et al.* [12], a layer by layer cationic nanostructures was fabricated on silicon surfaces to eliminate the bacteria and prevent the creation of biofilm successfully. These coating approaches have promoted ureteral stent properties to a certain degree.

Infection remains a major issue in the urinary system. It was usually resolved by the presence of an antibacterial agent, whereas this would easily result in drug resistance. Researchers have focused their attention on copper, which is an essential trace element in the human body that performs important biofunctions in physiological processes [13, 14]. The antibacterial characteristics of copper are attributed to disrupting the bacterial membranes, resulting in the microorganism's death, which is different from antibiotics [15]. In general, encrustation occurs coupled with infection as a result of an elevation in urine pH caused by urease-producing bacteria, which in turn causes the deposition of mineral crystals onto the surface of a ureteral stent. However, it cannot be only prevented by copper [16]. In a study by Jones *et al.*, the effects of artificial urine (AU) components and inhibitory agents against the action of urease on encrustation were investigated. It was revealed that a change in the calcium and magnesium salts ratio and albumin concentration altered the mass of encrustation. Moreover, the surface properties with good hydrophilicity, stable zeta potential and the presence of urease inhibitors significantly produced promising ureteral stents [17, 18].

Glycosaminoglycans are a common constituent of human urine and have been reported to be natural inhibitors of crystal formation by binding to urinary components and blocking the sites that are associated with crystal growth, which can be beneficial for minimizing stent encrustation [19, 20]. Heparin, which is one of the glycosaminoglycans, has shown to possess the strongest inhibitory effect due to its high electronegativity (-${\text{OSO}}_{3}^{\;-}$, -COO^−^) that repels cellular organisms [21–23]. The negative charge from the sulfate group and uronic acid of the heparin molecule interacts with Ca^2+^, which can prevent nucleation, growth and aggregation of crystals [24]. Electrostatic binding is a technique used to incorporate heparin onto biomaterial surfaces. Poly-L-lysine (PLL) has been designed to serve as functional biomedical materials where the activity is due primarily to their cationic nature. On the basis of electrostatic interactions, PLL is commonly used for loading of negatively charged biomolecules.

Thus, in the present study, heparin/poly-L-lysine-copper (Hep/PLL-Cu) nanoparticles (NPs) were prepared and immobilized onto PU by ‘dip-coating technique’. X-ray photoelectron spectroscopy (XPS), atom force microscopy (AFM), contact angle measurement, quantity of immobilized heparin and copper release were performed to characterize the coating properties. Both antibacterial rate and encrustation rate were evaluated and a short-term *in vivo* test was performed to investigate the antibacterial and anti-encrustation potencies of the coating.

## Materials and experiments

### Materials

PU was purchased from Lubrizol Corporation, USA. Dopamine hydrochloride, Tris base, toluidine blue O (TBO), and copper chloride (CuCl_2_) were supplied from Sigma-Aldrich. Heparin Sodium (Hep, MW< 8KDa) and PLL (MW 150–300 kDa) were purchased from Shanghai Bioscience and Technology Company. Dulbecco’s modified eagle medium (DMEM) and fetal bovine serum (FBS) were purchased from Gibico. All the additional reagents and solvents were of high analytical grades and commercially available.

### Nanoparticles preparation and immobilization

**Figure 1.**
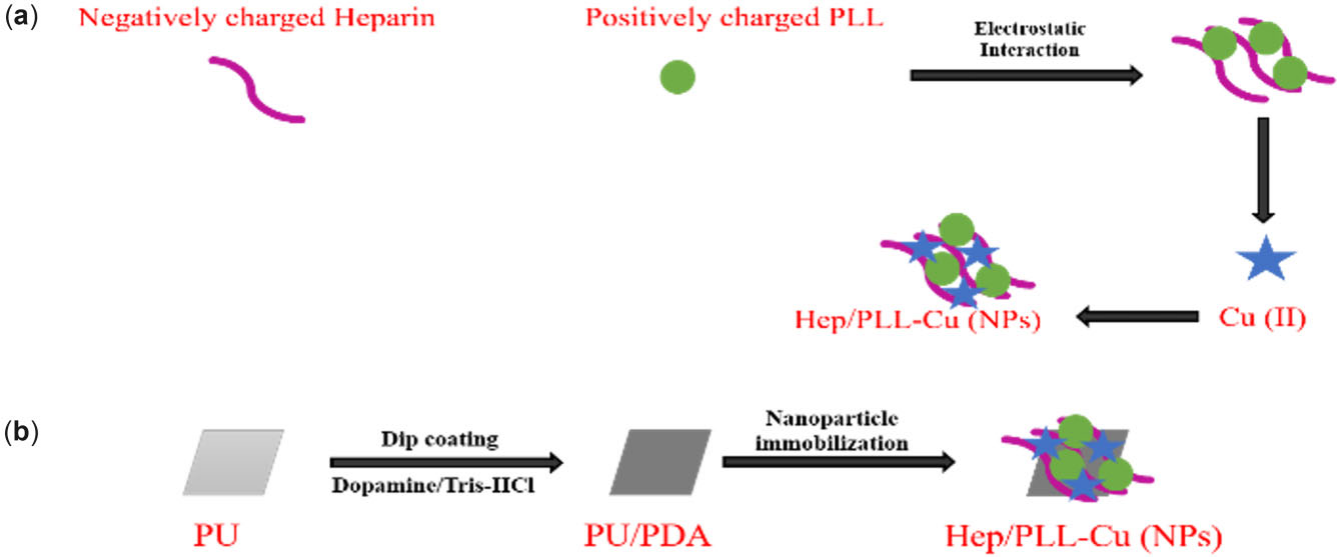
Schematic drawing of the preparation and immobilization process of NPs on PU/PDA.

Figure 1 shows the fabrication process of Hep/PLL-Cu NPs and immobilization on PU substrate. Firstly, PU substrates were cut into certain shapes (10 mm × 10 mm), followed by ultrasonic cleaning with water and ethanol for 10 min, respectively. Subsequently, the cleaned substrates were immersed into 2 mg/ml dopamine solution (10 mM Tris buffer, pH = 8.5) at 20°C for 12 h. Afterward, the substrates were rinsed twice with deionized water for 5 min, air-dried and labeled as PU/PDA (Polydopamine). An equal volume of Hep solution (5 mg/ml) and PLL solution (1 mg/ml) were mixed in the ultrasonic condition for 10 min. Then, the Hep/PLL nanoparticle solution was added to 10 ml of CuCl_2_ solution (2 mg/ml). Subsequently, the prepared NPs were immobilized on PU/PDA at 20°C for 24 h with gentle shaking. Finally, the constructed immobilized substrates, which were named Hep/PLL-Cu (NPs), were rinsed with deionized water three times.

### AU preparation

The AU was made based on previously reported composition [25], which contained the appropriate amount of inorganic human urine. The pH was adjusted to 6.4. Table 1 shows the listed AU reagents.

**Table 1.** Composition of AU

Reagent	Mass (g)^a^
NaCl	6.17
NaH_2_PO_4_	4.5
Na_3_C_2_H_5_O_7_	0.944
MgSO_4_	0.463
NaSO_4_	2.408
KCl	4.75
Na_2_C_2_O_4_	0.043
CaCl_2_	0.638

a One liter of distilled water.

### Nanoparticles morphology and size

The mean size, poly dispersion index (PDI) together with Zeta potential of the NPs dispersed in Tris buffer solution were determined by dynamic light scattering (DLS) using a Zetasizer Nano-ZS90 (Malvern Ltd., Malvern, UK). The morphology of the NPs was determined by transmission electron microscopy (TEM, FEI-F20, USA). Ten microliters of the purified TEM sample was placed on a copper grid with a carbon-coated Formvar film. After the solution was deposited, a strip of filter paper was used to remove the excess solution. Following this, the sample was dried at room temperature and examined by TEM.

### Scanning electron microscopy and AFM observation

The morphology of the coatings was examined by a scanning electron microscopy (SEM, JSM-6301 F, Japan). Besides, the changes of surface topography and roughness before and after NPs immobilization were characterized by AFM (SPA400, SEIKO, Japan). Image analysis was performed using the CSPM Image software.

### XPS analysis

XPS (XSAM800, Kratos Ltd, UK) with an Al Ka X-ray source (1486.6 *eV*) was used for elemental composition measurement.

### Contact angle measurement

Surface hydrophilicity was evaluated on a contact angle goniometer (JC2000C, Shanghai Zhongcheng, China) using the sessile drop method. There were two parallel samples in each group, and for each sample, measurements were taken at three random sites to obtain the average and standard deviations.

### Film thickness

The film thickness was measured by an ellipsometer (M-2000 DITM, J.A. Woollam) using the Cauchy model at a static angle of 60° and wavelength of 658 nm.

### Heparin and Cu ions releasing test

Each sample was immersed in AU at 37°C and shaken at 60 rpm for 1, 7, 14, 21 and 28 days in an air-tight centrifuge tube. A ratio of samples’ surface area to volume of 3 cm^2^/ml was used according to ISO 10993-12. The AU solution was changed daily, and the release medium at each specific immersion time was collected for both heparin and Cu ions releasing. The release rate of heparin was determined by a slightly modified TBO assay, which was measured at 530 nm by a microplate reader (Quant, Bio-Rad, USA) and evaluated by a calibration standard curve [26]. The released Cu ions concentration in the solution was detected by inductively coupled plasma-mass spectrometry (ICP-MS, 7900 Agilent Technologies). There were four parallel samples in each group.

### Cells culture and *in vitro* cytotoxicity assay

The human urethral epithelial cell (HUEC) lines were obtained from the Type Culture Collection of the Chinese Academy of Sciences, Shanghai, China, and cultured at 37°C with 5% CO_2_. For extracts preparation, samples were immersed in DMEM solution with 10% FBS containing 100 μg/ml penicillin and 100 μg/ml streptomycin for 24 h. One hundred microliters cell suspension with a density of 2 × 10^4^ cells/ml was seeded in the 96 well plates and incubated for 24 h to allow the attachment. The medium was then replaced with 100 μl extracts or DMEM (negative control). After 1, 3 and 5 days of incubations, 10 µl MTS (3-(4,5-dimethylthiazol-2-yl)-5-(3-carboxymethoxyphenyl)-2-(4-sulfophenyl)-2H-tetrazolium) was added and incubated for 3 h. The optical density (OD) was tested at 490 nm. The relative growth rate (RGR) was calculated by the following equation:

(1) $$\mathrm{RGR \% =}\frac{{\text{OD}}_{sample}}{{\text{OD}}_{negative}}\times 100\%,$$

where OD_sample_ is the optical density for PU, NPs or PU/PDA, while OD_negative_ represents that of negative control.

### Antibacterial test

*Proteus mirabilis*, a gram-negative bacteria strain associated with urinary tract infection, was obtained from Guangdong Microbiology Culture Center (Guangzhou, China). Samples were immersed in 75% ethyl alcohol for sterilization. Then, 50 µl bacterial suspension, which was diluted to 10^5^ CFU/ml in the AU solution, was added to the surface of the samples. After incubation for 24 h, the samples with bacterial suspension were transferred into a centrifuge tube with 2 ml AU solution and vortexed for 1 min to dilute and dislodge the bacteria sufficiently. Subsequently, 100 μl of the diluted bacterial solution was pipetted onto the solidified nutrient agar plates in triplicates and incubated overnight at 37°C. The bacterial colonies were counted and the antibacterial rate was calculated as below:

(2) $$\mathrm{Antibacterial}\;\mathrm{rate}\;\left(\%\right)=100\%\;\times (A-C)/A,$$

where A and C represent the blank group and C represents the experimental samples (PU, PU/PDA and NPs).

### Live/dead staining assay

Samples were incubated with 1 ml bacterial suspension (3 × 10^8^ CFU/ml) for 24 h to allow the adherence of bacteria on the surface. After rinsing with AU solution to disentangle any bacterial loosely hanging, 100 µl mixed solution (SYTO-9 dye: PI = 1:1) was added according to the instruction of the manufacturer: Live/Dead BacLight Bacterial Viability Kit (Invitrogen Molecular probes, Darmstadt, Germany). A fluorescence microscope (BX53, Olympus, Japan) was implemented to observe the biofilm characteristic.

### *In vitro* encrustation analysis

Ten parallel samples of each group were suspended in the AU solution in 15 ml Eppendorf tubes and encrusted for 2, 4 and 6 weeks, respectively. Ten milliliters of AU was replaced daily to stimulate physiological conditions in the urinary tract and maintained in an incubator at 37°C. Following the specific immersion time, the samples were taken out and rinsed in deionized water. After drying, the weight increase was measured. The encrustation ability of the samples was characterized by the encrustation mass per cm^2^, and calculated by the following equation:

(3) $$\text{Encrustation ability}\;\left(\text{mg}/{\text{cm}}^{2}\right)=\left(A_{i}{}_{}–A_{0}\right)/S,$$

where *A_i_* is the mass at the respective time points (2, 4 and 6 weeks), *A_0_* is the initial mass and *S* is the total surface of a sample. Furthermore, the encrusted Ca and Mg salts were dissolved in 5% HCl for 30 min and were measured by inductively coupled plasma optical emission spectrometry (ICP-OES, 5110, Agilent, USA). In addition, SEM (JSM-6301 F, Japan) coupled with energy spectroscopy (EDS, EDAX 9100, Japan) was used to characterize the surface morphology and the composition of encrustation.

### *In vivo* encrustation analysis

Clinical double J stent was cut into a size of 3 mm in length. NPs-loaded stent (coated stent) was treated using the same method with PU. All the animal experiments were conducted according to the approval of the Institutional Animal Care and Use Committee (IACUC) in Liaoning Changsheng Biotechnology Co. Ltd (Approval No. CSE202109001). Six Wistar rats (8–10 weeks old, male) weighing between 280 and 20 g were used and divided into the coated and uncoated groups (three in each group). After interperitoneal anesthesia of sodium pentobarbital (30 mg/kg), a stent was inserted into the rat’s bladder. At the same time, 0.2 ml *P. mirabilis* at a concentration of 3 × 10^6^ CFU/ml was injected and kept for 10 min. After feeding for 7 days, the rats were sacrificed and stents were removed. After air-drying for 48 h, the surface morphology and composition of encrustation were evaluated by SEM equipped with EDS. Following this, ICP-OES measurement of Ca and Mg salts was used to quantitatively analyze the encrustation as a supplement to the observation of surface morphology.

### Statistical analysis

The results were expressed as mean ± standard deviation and one-way analysis of variance was conducted by Origin 9.1 software to evaluate the difference between data sets. *P <* *0.05 was shown as a significant difference, and △*P *≥* *0.05 was no significant difference.

## Results

### Size and zeta potential of NPs

The particle size, PDI and Zeta potential of the NPs were obtained by DLS as shown in Table 2. The mean size of NPs is 345 ± 8.1 nm, supported by a Zeta potential −30.9 ± 0.97 mV. Alongside the PDI value is 0.137.

**Table 2.** Properties of NPs

Description	Size (nm)	PDI	Zeta potential (mV)
Hep/PLL-Cu	345.5 ± 8.1	0.137	−30.9 ± 0.97

### TEM observation

**Figure 2.**
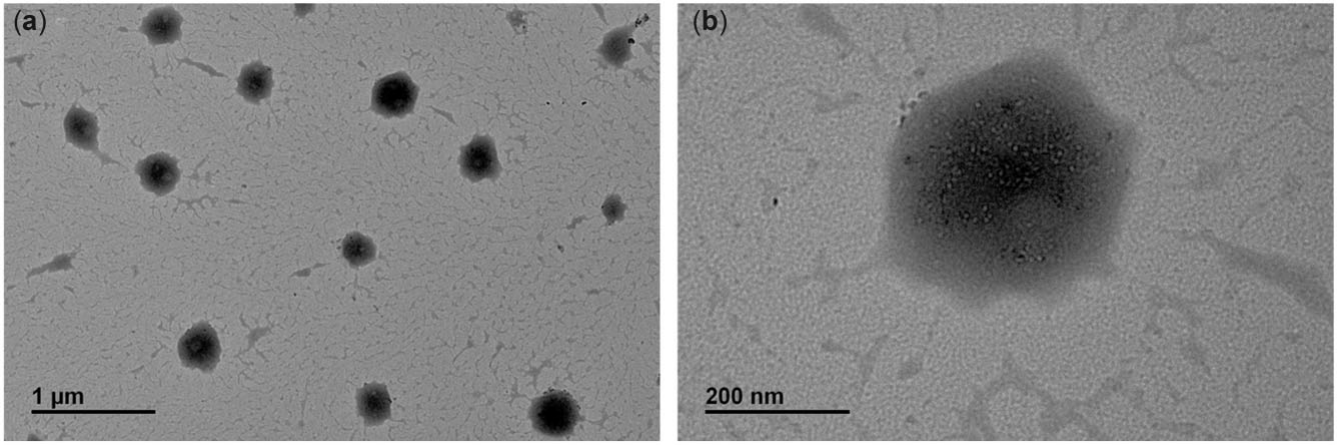
TEM images of prepared nanoparticle.

The TEM images in Fig. 2a and b demonstrated that the NPs were successfully prepared and mostly spherical. The size was also in a nanoscale range.

### SEM observation

**Figure 3.**
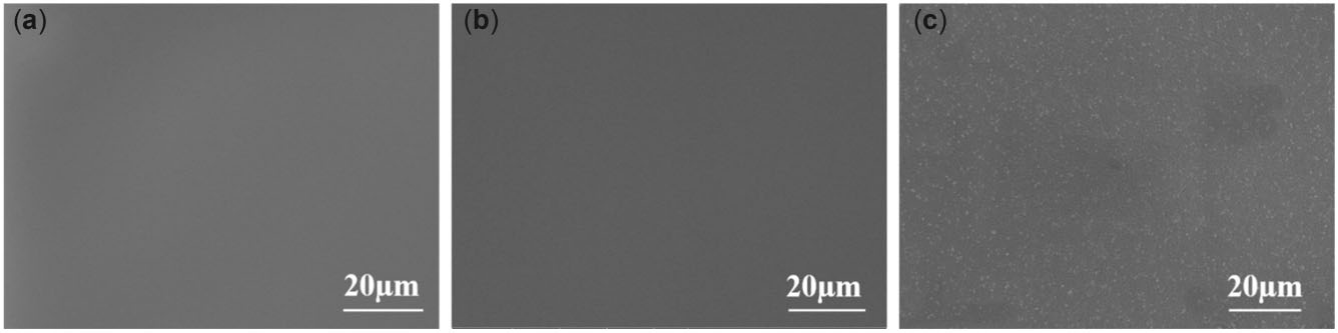
SEM images for (**a**) PU, (**b**) PU/PDA, (**c**) NPs.

As shown in Fig. 3a and b, the PU and PU/PDA revealed a smooth surface. After NPs immobilization in Fig. 3c, there appears very tiny aggregates on the surface. The change in the surface topography demonstrated a uniform distribution of the nanoparticles.

### AFM observation

**Figure 4.**
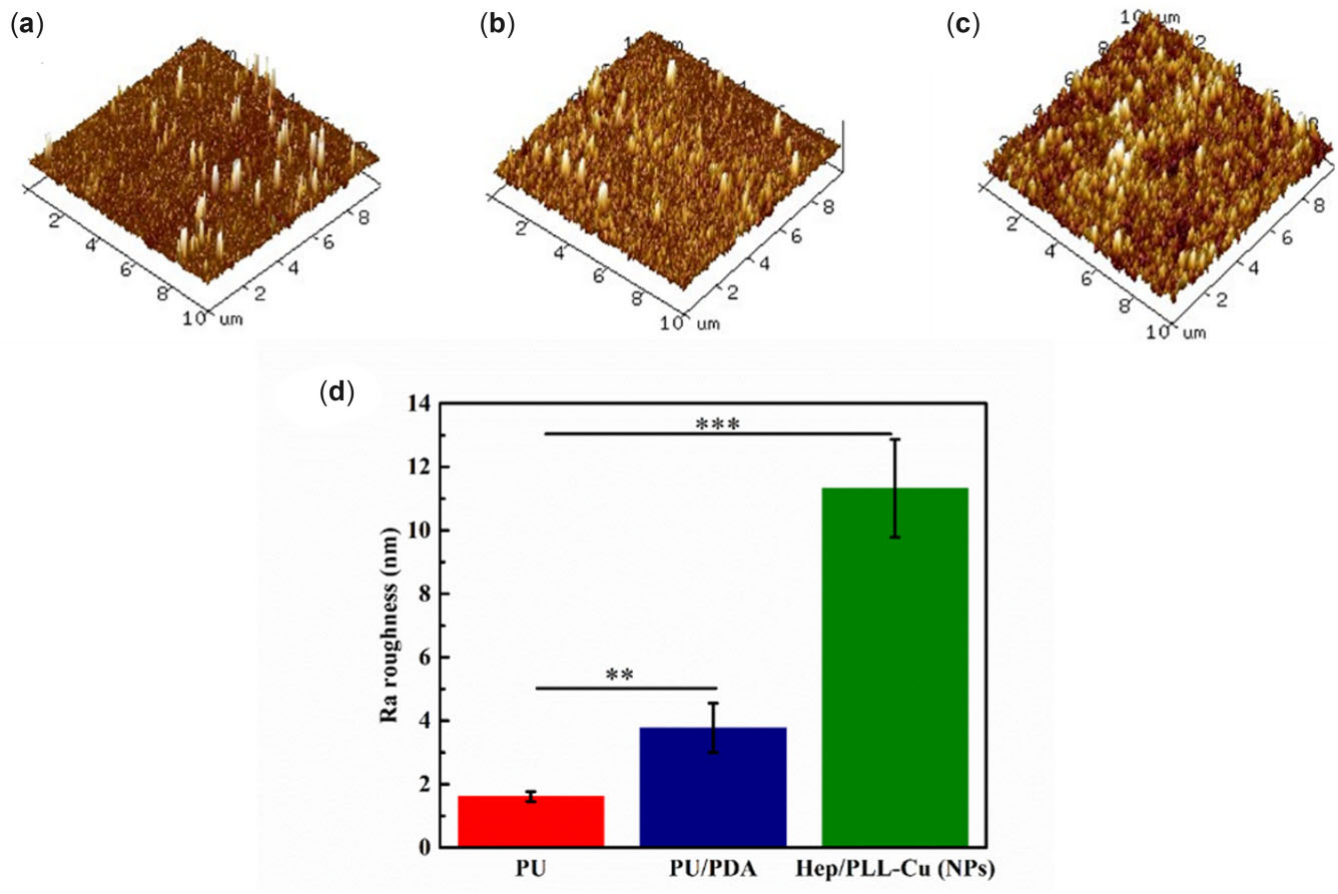
(**a–c**) 3D dimensional AFM images for PU, PU/PDA and NPs; (**d**) surface roughness of samples, data were presented as mean and standard deviation (*n* = 3, **P <* *0.01, ***P <* *0.001).

Figure 4 shows the 3D tomographic profile and roughness (Ra) in bar chat, respectively. The PU surface reveals the smallest roughness, Ra =1.61 ± 0.15 nm compared with 3.77 ± 0.77 and 11.32 ± 1.54 nm for PU/PDA and NPs, attributing to the deposition of PDA aggregation and immobilization of nanoparticles.

### XPS analysis

**Figure 5.**
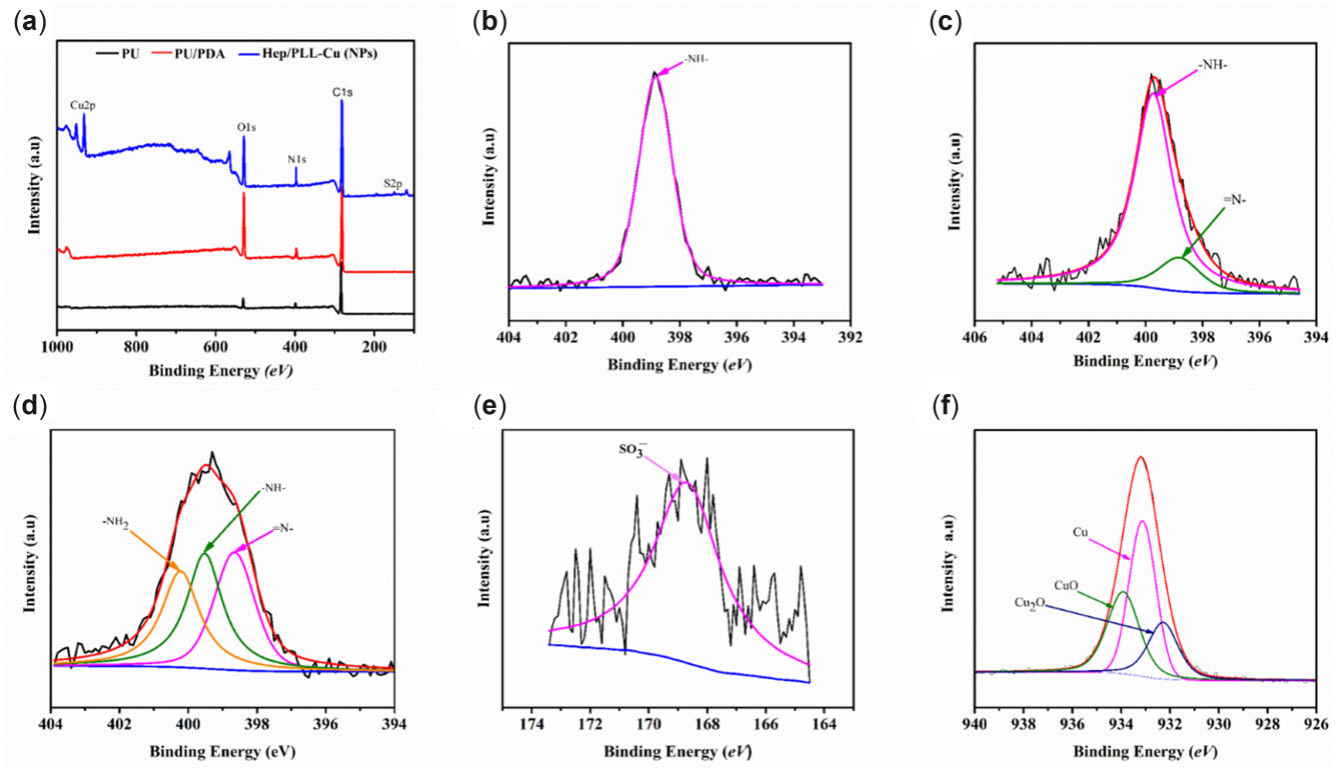
(**a**) XPS wide scan of spectra; (**b**) PU high-resolution spectra of N1s; (**c**) PU/PDA high-resolution spectra of N1s; (**d**) NPs high-resolution spectra of N1s; (**e**) S2p high-resolution spectra; and (**f**) high-resolution spectra of Cu2p.

The XPS wide-scan spectra of the samples with their respective surface elemental compositions are shown in Fig. 5a. To confirm a successful PDA coating on PU, the N1s high-resolution XPS curve fitting was performed on PU and PU/PDA. A secondary amine (-NH-) with a binding energy of 399.5 *eV* appeared in PU, while an additional amino rich tertiary group (=N-) with binding energy of 398.5 *eV* appeared in PU/PDA, as shown in Fig. 5b and c, respectively. After nanoparticles immobilization, -NH_2_ was presented, which was the characteristic peak of heparin and PLL (Fig. 5d). Meanwhile, the nitrogen content increased to 8.14% on NPs surface compared to that on PU/PDA surface (6.38%), which was also ascribed to the amine group in heparin and PLL, as shown in Table 3. To investigate the specific composition of S2p and Cu2p, a high-resolution XPS curve fitting was performed. Regarding S2p, a sulfo group (168.5 *eV*) was detected and ascribed to the presence of heparin in Fig. 5e. The spectra of Cu2p are shown in Fig. 5f, and the XPS data indicate the existence of Cu (932.5 *eV*), CuO (933.6 *eV*) and Cu_2_O (932 *eV*).

**Table 3.** Elemental compositions determined by XPS

Sample	C%	N%	O%	S%	Cu%
PU/PDA	73.4	6.38	19.84		
Hep/PLL-Cu	60.73	8.14	21.10	1.78	8.25

### Contact angle measurement

**Figure 6.**
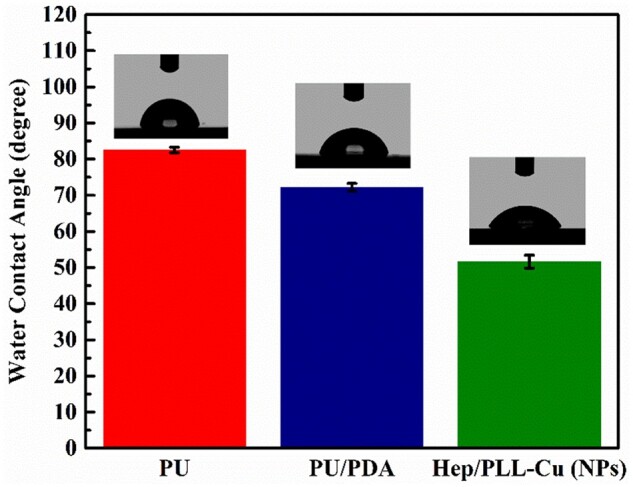
Water contact angles and droplet images of water.

Figure 6 depicts the mean water contact angles showing that PU was 82 ± 0.75° and it was reduced to 72 ± 1.39° on the PU/PDA. The water contact angle was decreased to 54 ± 1.54° after NPs immobilization.

### Film thickness

The coatings thickness of PDA and NPs was measured by an ellipsometer. As shown in Table 4, the film thickness increased from 5.82 nm for PU/PDA to 10.58 nm after the immobilization of NPs.

**Table 4.** Thickness of coatings

Surface Characteristics	PU	PU/PDA	NPs
Thickness (nm)	None	5.82	10.58

### Heparin and Cu ions release

**Figure 7.**
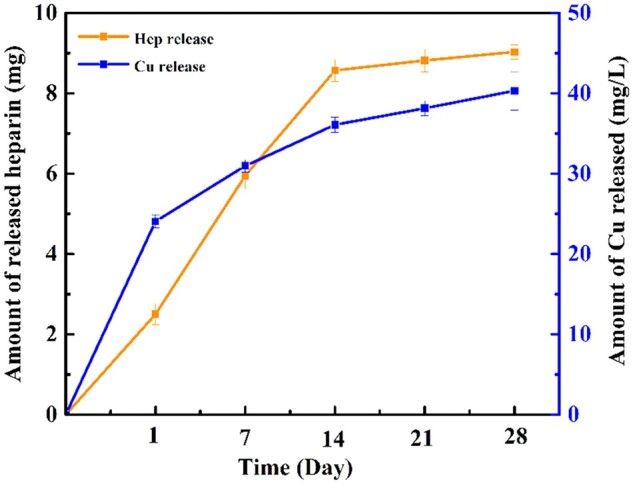
Cumulative releases of heparin and Cu ions from NPs immobilized surface (*n* = 3).

The TBO assay was used to study the progressive release quantity of heparin at each time point. As shown in Fig. 7, the nanoparticles immobilized surface displayed a small heparin release behavior within 24 h, and the cumulative release amount of heparin on day 14 reached to 8.57 mg. Subsequently, the heparin release curve tended to balance even after 4 weeks. The amount of Cu ions released by nanoparticles was also measured in AU solution. It was observed that the release of Cu ions increased with immersion time, and there was no burst release of Cu ions within 24 h. The cumulative release amount of Cu ions on day 14 reached 36 mg/l, and a steady release rate was maintained even after 4 weeks. The amount of released heparin was correlated with the Cu release.

### *In vitro* cytotoxicity

**Figure 8.**
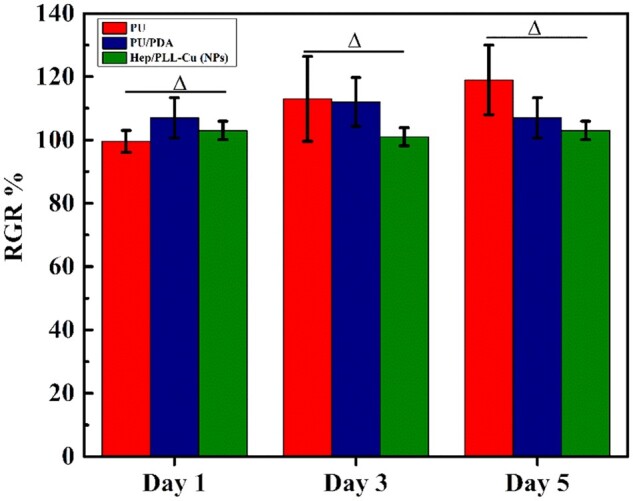
Cells proliferation rates of different samples cultured for 1, 3 and 5 days. Data were presented as mean and standard deviation (*n* = 3, △*P *≥* *0.05).

The proliferation activity of HUECs after cultivating with extracts was evaluated by MTS assay and the results are presented in Fig. 8. The results revealed a progressive proliferation rate during the culturing time from day 1 to day 5. Besides, the RGRs were all greater than 85%, demonstrating no cytotoxicity.

### Antibacterial activity

**Figure 9.**
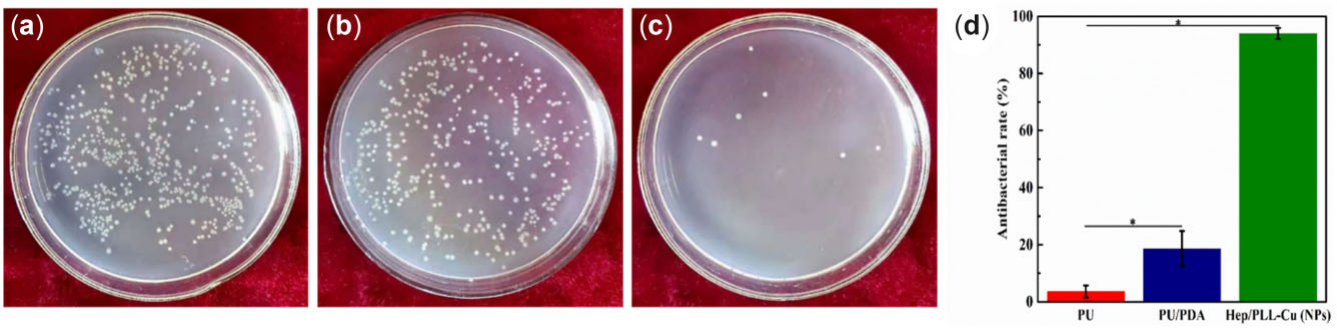
Bacterial colonies counts in the AU solution after incubating with different samples for 24 h, (**a**) PU, (**b**) PU/PDA, (**c**) NPs, (**d**) antibacterial rates of different samples in AU solution. Data were presented as mean and standard deviation (*n* = 3, *P <* *0.05).

Figure 9 presents the antibacterial potency of the samples cocultured with *P. mirabilis* suspension in AU solution for 24 h. A substantial number of bacterial colonies were observed on PU and PU/PDA. However, NPs showed a significant reduction in bacterial colonies, showing antibacterial performance. The antibacterial rate of NPs was over 90%, which was much higher than that of PU and PU/PDA.

### Bacterial staining assay

**Figure 10.**
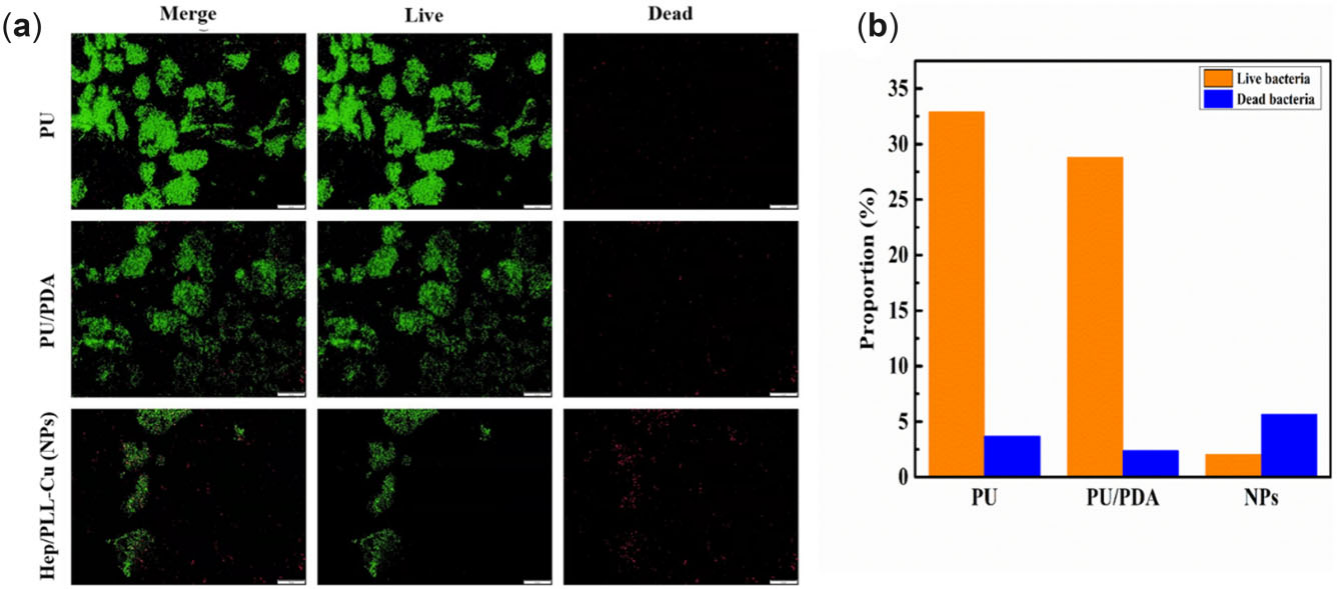
(**a**) Merge fluorescent images indicating live and dead bacteria on samples after 24 h coculture, (**b**) proportion of live and dead bacteria.

The inhibitive ability against adhesion and colonization of bacteria on samples was evaluated by live/dead staining assay. The live bacteria appeared in green, whereas the dead bacteria stained in red. Figure 10 shows that the adhered bacteria on PU and PU/PDA formed a biofilm, which could possibly host the bacteria. On the contrary, the bacteria on the NPs appeared scattered and a significant number of them were killed indicating potential antibacterial efficiency.

### *In vitro* encrustation analysis

**Figure 11.**
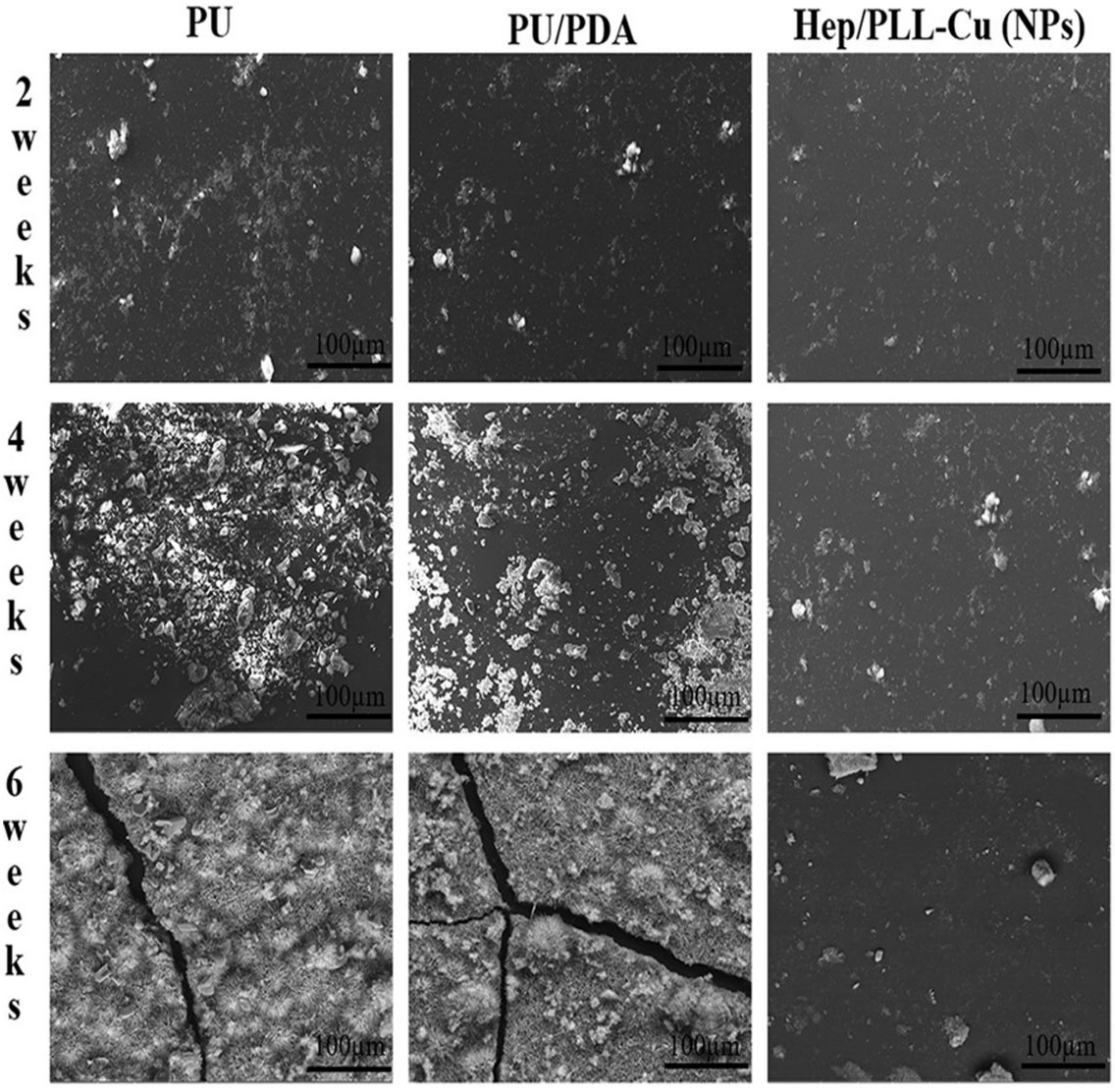
SEM micrographs of different samples immersed in AU solution for 2, 4 and 6 weeks.

The appearance of urinary encrustation on surfaces of the samples was examined by SEM. As shown in Fig. 11, encrusted crystals were rarely visible on the samples at 2 weeks. After 4 weeks of immersion, encrustation was deposited on the majority of PU and PU/PDA. Furthermore, it can be seen that the surfaces of PU and PU/PDA were fully covered at 6 weeks. However, NPs exhibited much less amount of encrustation.

**Figure 12.**
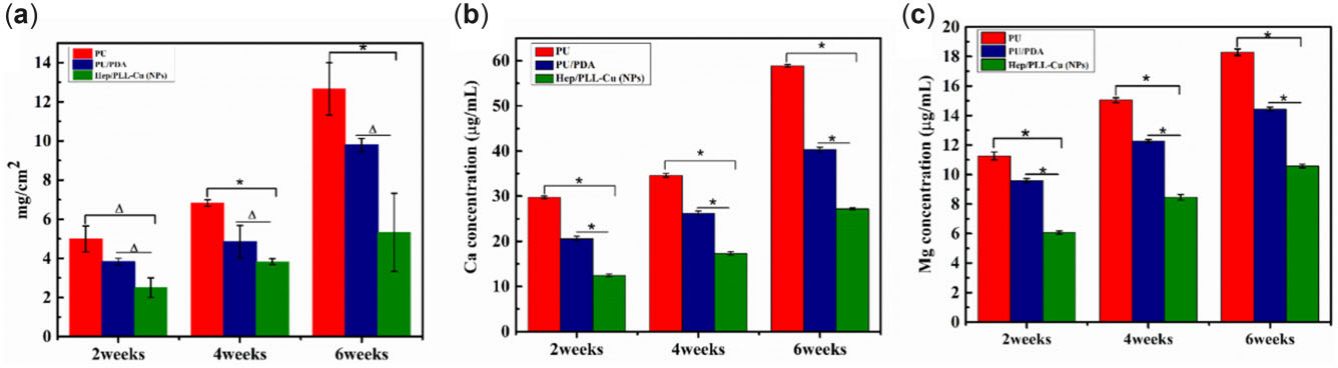
(**a**) Encrustation weight, Ca (**b**) and Mg (**c**) contents of different samples after immersions in AU solution for 2, 4 and 6 weeks, *P ≤* *0.05, △*P *≥* *0.05.

Figure 12 depicts the weight change per cm^2^ of encrustation in response to immersion time. The encrustation rates of nanoparticles were 1.88, 4.19 mg/cm^2^ and 5.86 g/cm^2^ after 2, 4 and 6 weeks immersions, respectively, which were lower than that of PU/PDA or PU at any time point. For further evaluation, contents of Ca and Mg ions in encrustation that was dissolved in 5% HCl solution were measured by ICP, as shown in Fig. 12b and c. The contents of NPs, not only Ca but also Mg, were all significantly lower than those of PU and PU/PDA, revealing a property of encrustation resistance.

### *In vivo* encrustation analysis

**Figure 13.**
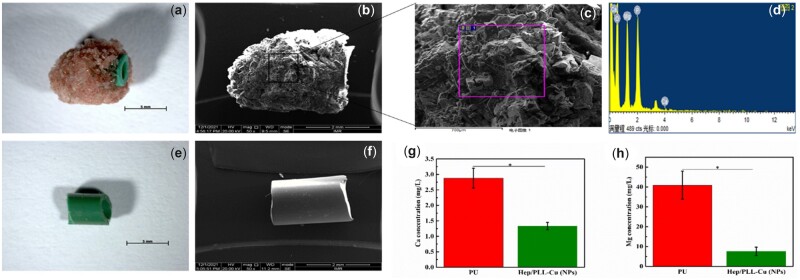
Encrustations of the ureteral stents after 7 days implantation, (**a**) photo of implanted PU stent, (**b**) SEM image of implanted PU stent, (**c**) SEM micrograph of encrustation, (**d**) EDS spectra at encrustation site, (**e**) photo of implanted nanoparticles stent, (**f**) SEM image of implanted nanoparticles stent, Ca (**g**) and Mg (**h**) deposition amounts on nanoparticles and PU ureteral stent after 7 days of implantation (*n* = 3, *P <* *0.05).

Figure 13 presents the SEM micrographs of the NPs coated stent and bare PU stent. It can be seen that there was almost no encrustation after grafting with NPs, while the bare PU revealed a large amount of encrustation. The Ca and Mg ions depositions on the implanted stents were quantitatively measured by ICP-OES. The results showed a lower encrustation of Ca and Mg ions of NPs compared to PU.

**Figure 14.**
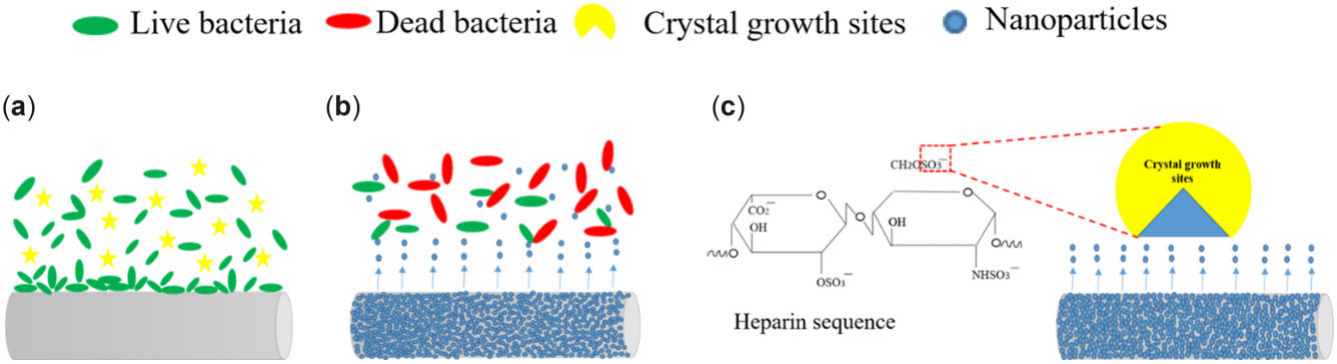
Mechanism of Hep/PLL-Cu (NPs) stent, (**a**) PU stent, (**b**) release of Cu ions from the NPs killing majority of the urease-producing bacteria on NPs, (**c**) release of ${\text{SO}}_{3}^{-}$ group from heparin molecule blocks the crystal growth sites hereby reducing the formation of urine crystals.

## Discussion

The obstruction of the ureter is a significant problem in the urological field. The fundamental function of the ureter stent is to open the obstruction and to aid urine flow. Patients with strictures or obstructions from urinary stones need to be drained by ureteral stents to avoid hydronephrosis and renal failure [27]. Unfortunately, the presence of biofilm formation and encrustation constantly blocks the flow of urine. To overcome these challenges, multifunctional surface modification of ureter stents has been a concern. In the present study, the crystallization inhibiting and antibacterial effects of heparin and Cu, which were coated on ureteral stent, were demonstrated. The heparin coupled with Cu nanoparticles was produced by an electrostatic interaction of negatively charged Hep and positively charged PLL and Cu ions. The NPs were immobilized on the stents by PDA, which had been reported to have the tendency of creating a dense coating on substrates by self-polymerization and to be active for biomolecules with amine groups [28].

Following preparation, analysis of NPs was performed to ensure their suitability by *in vitro* and *in vivo* experiments. The particle size, homogeneity and stability of NPs have a direct influence on the immobilization profile of the nanoparticles on the dopamine-coated surface. These parameters are essential for the interactions of NPs with living cells and possess active reaction sites on the surfaces of biomaterials. Regarding the particle size distribution and Zeta potential as shown in Table 2, the particle dispersion index (PDI) values of 0.2 and below, as a measure of the size distribution of particles, were suitable for polymer-based nanoparticle materials [29, 30]. Liu *et al.* [31] found that a smaller PDI value indicated a better uniformity and enhanced electrostatic interactions between nanoparticles. It is well known that the Zeta potential reveals the specific surface charge properties of a nanoparticle suspension, which depicts the potential stability of the nanoparticles. It was reported by Kumar and Dixit [32] that Zeta potential values of −30 mV and above showed moderate stability of nanoparticles. TEM results in Fig. 2 showed the spherical morphology of the NPs and proved the NPs to be in nanoscale range. The SEM images in Fig. 3 revealed a uniform distribution of the NPs, indicating a successful coating immobilized. Furthermore, AFM images in Fig. 4 also indicated homogenous distribution of NPs, suggesting that the immobilization of NPs possessed adequate stability. To further confirm the successful PDA and NPs coating, high-resolution spectra of N1s were measured. The presence of a secondary and tertiary amine group of PU/PDA with binding energies of 399.5 and 398.5 *eV*, as shown in Fig. 5c, indicated successful PDA deposition. Comparison between PU/PDA and NPs,-NH_2_ was observed on NPs, attributing to heparin and PLL, which depicted that the NPs had been conjugated onto PU. Meanwhile, the increased content of nitrogen was also due to the heparin and PLL coating. From XPS spectra recorded in Fig. 5e and f, there were two new peaks: S2p (168.5 *eV*) and Cu2p (932.4 *eV*) identified on the NPs spectrum. The S2p peak (Fig. 5e) was an indication of the successful immobilization of heparin by the presence of the sulfate group (${\text{SO}}_{3}^{\;-}$) in the heparin backbone [33]. Furthermore, the Cu2p spectra in Fig. 5f revealed three main peaks with binding energies of 932.5, 933.6 and 932 *eV* corresponding to Cu, CuO and Cu_2_O, respectively, which exhibited antibacterial activity. Besides, the surface hydrophilicity of a ureteral stent plays an important role in promoting or inhibiting bacterial adhesion [34]. Heparin and PLL are abundant in hydrophilic groups such as amine, carboxyl, hydroxyl and sulfo groups [31, 35], as a result of the nanoparticle immobilization, the contact angles were reduced.

One of the most essential properties for biomaterials is biocompatibility, which is directly affected by the stability of the nanoparticles on the dopamine-coated surface. An initial burst release is considered to be harmful, and it can sometimes have detrimental consequences. The burst release of heparin may increase the risk of bleeding while Cu can result in liver and kidney damages. In ensuring that the nanoparticles sustained release and did not result in cell toxicity, the heparin and Cu ions release contents, and the effects of the nanoparticles on the proliferative activity of HUECs were assessed. According to the heparin and Cu ions release curves shown in Fig. 7, there was no burst release of either heparin or Cu ions in the beginning. As the immersion time increased, it was observed that after day 14, there was a steady release of heparin and Cu ions, which suggested a good biological activity of the coatings. Furthermore, the RGRs evaluated by MTS assay were all over 85%, indicating no cytotoxicity with increasing the time of cultivation.

In general, bacteria cling to the catheter surface via adsorbed molecules, and the adherent bacteria create a biofilm after catheterization. The formed biofilm protects the bacteria against antibiotics and antibodies, which makes it a significant problem. Hence, if the adherence of uropathogens is controlled, the infection may be prevented. As the plotting in Fig. 9, the addition of Cu into the nanoparticles significantly reduced the bacterial proliferation on the surface by continuous release of Cu ions, which damaged the bacterial cell wall, resulting in cell death [36]. Besides, the inhibiting of *P. mirabilis* could decrease the encrustation deposition. *P. mirabilis*, a representative urease-bacteria, secreted urease and reacted with urea to form ammonia and CO_2._ The release of Cu ions from the NPs decreased the urease amount produced by the bacteria, which in turn restrained the elevation of urine pH and retarded the precipitation of Ca and Mg phosphate crystals on the catheter [37].

Although bacterial biofilm and crystalline deposits are inextricably linked, the surface property of a stent is also crucial to the encrustation. Glycosaminoglycans have been detected on urinary tract tissue surfaces to prevent bacterial, protein and ionic adhesions to the cell membrane [38]. Therefore, heparin, one kind of glycosaminoglycans, has been focused on its potential to inhibit the encrustation by acting as a crystal growth inhibitor. The formation of crystals depends on a sequence of nucleation, growth and aggregation [39, 40]. When the sulfate group and uronic acid in the heparin molecule bind to the urine components, it blocks the crystal growth sites in the epithelial cells, urinary cast and red blood cells [41]. As a result, the blocked urine components are excluded from crystallization processes in the urinary tract. Moreover, heparin can protect the mucosa of the urinary tract, which prevents crystal adherence [24]. Figure 11 depicts that the encrustation formed on NPs samples was much less than that on PU and PU/PDA, indicating that heparin had effects on preventing crystalline deposition.

The *in vitro* results were not conclusive evidence, it is necessary to confirm the NPs coatings *in vivo* for further verification on the ureteral stent [42, 43]. After 7 days of implantation, the PU stent showed severe encrustation, whereas the NPs stent indicated no considerable encrustation as seen in Figure 14. Urine pathogens affect the parameters and composition of the urine, which should affect the formation of urinary stones [44]. The composition of encrustation formed on the PU stent includes struvite (NH_4_MgPO_4_.2H_2_O), hydroxyapatite (Ca_5_(PO_4_)_3_(OH)) and brushite (Ca(HPO_4_)·2H_2_O) [45], attributing to urea hydrolysis to ammonia and bicarbonate. In our next work, a longer *in vivo* test will be done to investigate more on the NPs ability to inhibit both bacterial biofilm and encrustation.

## Conclusion

In this study, a novel anti-bacterial and anti-encrustation coating was developed to relieve the complications of urinary catheters. Hep/PLL-Cu (NPs) were prepared and immobilized on a polydopamine coated PU. The *in vitro* study results showed that the NPs had good chemical surface characteristics (surface charge and hydrophilicity), no toxicity, and potency to inhibit bacterial adhesion, as well as Ca and Mg deposits (encrustation). In addition, the *in vivo* results demonstrated that the NPs coating on PU stents significantly reduced the encrustation formation.

## Funding

Liaoning Science and Technology Program (grant No.2020JH2/10300159).

*Conflicts of interest statement.* The author(s) declared no conflicts of interest with respect to the research, authorship and publication of this article.
